# Rapidly enlarging axillary cystic hygroma in a 6-year-old male patient: A case report and literature review

**DOI:** 10.1016/j.ijscr.2022.107806

**Published:** 2022-11-30

**Authors:** Layth Al-Karaja, Mohammad G. Ibdah, Salem M. Tos, Narmeen Giacaman, Ahmadullah Musleh Abu Yousef, Rafiq Salhab

**Affiliations:** a Al-Quds University, College of Medicine, Palestine; b General Surgery Department, Al Ahli Hospital, Hebron, Palestine

**Keywords:** Axillary mass, Cystic swelling, Hygroma, Case report

## Abstract

**Introduction:**

Cystic hygromas are relatively uncommon tumors of lymphatic origin, it appears most of the time, approximately 90 % before age of 2 years, and it is caused by abnormal development of lymphatic vessels.

**Case presentation:**

Here we report a case of axillary cystic hygroma in a 6-year-old healthy boy, which presented with the rapid development of a right axillary mass during 3 days, without any predisposing factor.

**Discussion:**

Cystic hygromas occur due to complete or partial obstruction of lymphatic vessels, which leads to lack of communication with the venous system, this results in the accumulation of lymphatic fluid and swelling, it occurs in the cervicofacial region most of the time 75 %, but it can arise anywhere in the body, it classically presents as painless, soft mass, diagnosis can be done using ultrasound, CT, MRI, each of which has its advantages, surgical treatment is routinely favored, but other options are also available.

**Conclusion:**

Axillary cystic hygromas are quite rare, few cases have been reported in fetuses and adults, but only one case in the pediatric age group, thus, in light of these cases, cystic hygromas should be considered in the differential diagnosis of any cystic axillary swelling.

## Highlights

• The development of cystic hygroma in the axillary location in the pediatric age group is a quite rare condition that has been reported only a few times in literature.
• Cystic hygroma should be considered in the differential diagnosis of axillary swellings.
• Multiple treatment modalities have been described for this condition; surgery is still the treatment of choice.
• Surgical excision has been used in this patient.

## 1 Introduction

Cystic hygromas (CH) are uncommon tumors of lymphatic origin [2], which mainly occur in the first two years of life in more than 90 % of cases [4], they arise due to complete or incomplete obstruction of the lymphatic vessels which prevents the communication between the lymphatic and venous system causing the development of cysts, these cysts can be unilocular but more frequently multilocular, swelling of the cysts occurs due to lack of communication with the venous system, as a result, lymphatic fluid accumulates in them causing swelling [13]. CH is mainly located in the cervicofacial region, which accounts approximately for 75 % of cases [2], but it can occur anywhere in the body [10]. These tumors are classically present as soft, painless masses, usually transluminal, which often gives rise to patient worries.

The most favorable treatment for CH is still surgical, but other techniques have been described, they include sclerosing agents like doxycycline, radiation, cauterization, and aspiration [10].

Axillary location of cystic hygroma has been described multiple times in literature in the prenatal period and in adults [2], [3], [6], [11], [12], [13], [15], [16], [17], but only once in the pediatric age group, which were in a 5-year-old female child [14], so, to the best of our knowledge, this case should be the second.

Herein we report a case of axillary cystic hygroma in a 6-year-old healthy male child, which appeared within 3 days duration without any predisposing factor and was managed surgically.

This work has been reported in line with the SCARE criteria, which is used by authors, journal editors, and reviewers to increase the robustness and transparency in reporting surgical cases [18].

## 2 Case presentation

**Fig. 1:**
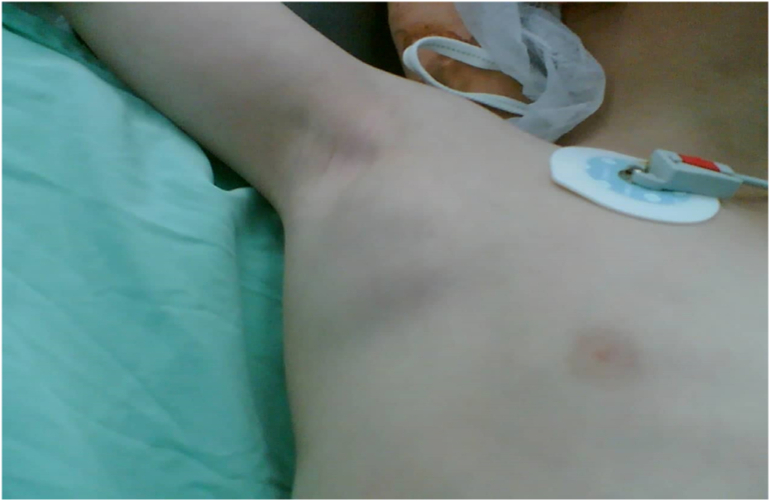
Photograph showing right axillary cystic hygroma.

A 6-year-old male patient presented to the hospital complaining of painless rapidly growing swelling in the right axilla that started 3 days before his admission with no history of trauma or infection, the swelling not associated with discharge, no erythema or discoloration, no fever, no dilated veins, no sinus tracts, and it's not expansible (Fig. 1).

On physical examination, the patient looks well, alert, and oriented, with only positive finding of a mass in the right axillary area with a size of 7 × 5 cm, globular in shape, soft in consistency, the mass is not tender, no warmth, it is movable with hand raising, not attached to the skin, no axillary lymphadenopathy.

Laboratory investigations revealed the following findings: WBC 9.2 K/μL, neutrophils 56 %, lymphocytes%, RBC 4.31 × 10^6 μL, Hb% 11.6 g/dL, HCT 34.3 %, MCV 79.5 μm^3, MCH 26.9 pg/mL, MCHC 33.8 %, RDW 12.8.

**Fig. 2:**
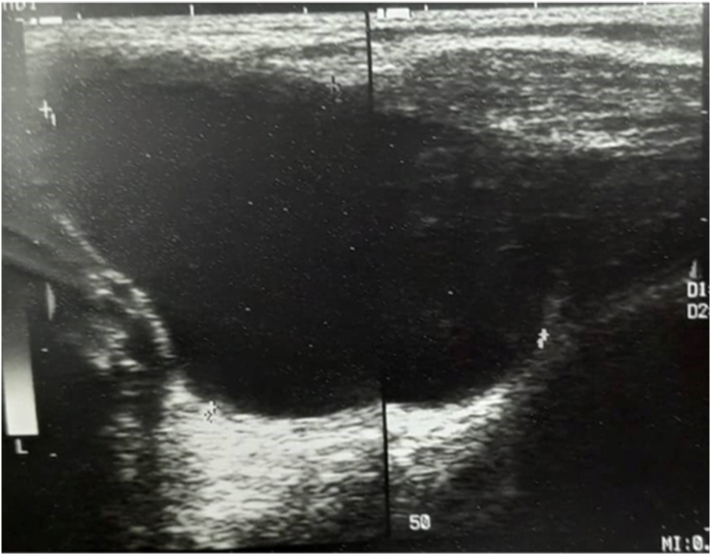
High linear frequency (10–15 MHz) ultrasound probe on a transverse cranial scan of the right axilla neck with a colour-Doppler module shows a hypoechoic unilocular cystic lesion with an absence of identifiable flow signal.

**Fig. 3:**
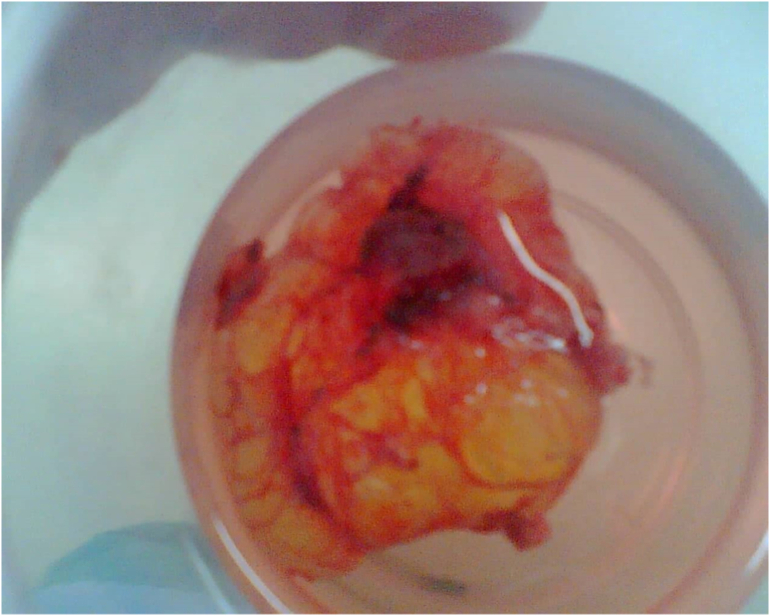
Specimen of the cystic hygroma post-operative.
The histopathology analysis found a cystic brownish mass measuring 8 cm in greatest dimensions, on incision it was filled with yellow fluid, and the wall thickness is up to 0.5 cm, in conclusion, the mass is consistent with cystic hygroma (also known as lymphangioma).

Right axillary ultrasonography findings (Fig. 2):

Revealed unilocular cystic mass in the right axilla measuring about 6 × 5.5 × 4 cm in maximal dimensions, located anterior to the axillary vessels, no septae, no calcifications or soft tissue component, no enlarged lymph nodes, no other abnormality.
The patient underwent surgical excision of the mass by right axillary transverse incision, opening all layers of the wall, identifying the mass, which was cystic in nature, then separated from all layers and excised outside in block (Fig. 3). All other structures remained intact, then all layers closed and the skin sutured, and the mass was sent to histopathology.

The patient improved after the surgery without any complications or recurrence in the follow-up.

## 3 Discussion

Cystic hygroma (CH), known also as cystic lymphangioma, is a benign congenital malformation of the lymphatic system that occurs as a result of the incomplete development of lymphatic vessels [1], it is an uncommon anomaly that occurs mainly in children and accounts for 6 % of all pediatric soft tissue tumors [2]. Cystic hygromas are mainly located in the cervical, axillary, inguinal, thoracic, and retroperitoneal regions [3], noting that almost 80 % of these lesions are found in the posterior triangle of the neck [5]. There are many papers about cystic hygromas, but only a few about axillary cystic hygroma.

Cystic hygromas are considered a tumor of the childhood period, as almost 90 % of cases occur before age of 2 years [4], development of CH at an older age can be related to the delayed proliferation of cell rests, or due to secondary predisposing factors like infections, trauma, or iatrogenic stimuli [6]. CH also can be associated with many syndromes, including Turner which is the most common syndrome to be associated with [12], others include Noonan and trisomies in general [5].

The classic presentation of cystic hygroma is a painless mass, which gives rise to worries of the parents about the lesion. Other presentations are related to the complications or effects of cystic hygroma -even though benign nature- such as respiratory distress, feeding difficulty, a sudden increase in the size, infection of the lesion with fever [10], and in a reported case, it can cause shoulder dystocia [11]. On physical examination, these lesions appear soft, compressible, non-tender, transluminal, and without bruits [10].

Differential diagnosis of the lesion includes hematoma, abscess or lymphocele formation, and soft tissue sarcomas [2]. Diagnosis is usually made by ultrasonography; it is particularly helpful in differentiating the solid or cystic nature of the mass. Typically, CH appears as multilocular cystic masses, containing septations of inconsistent thicknesses, while the fluid inside the cyst can appear as entirely anechoic, hypoechoic, or hyperechoic, this is based on the presence of infection, hemorrhage, or high lipid content [7].

Other diagnostic measures include CT and MRI, each of which has its advantages, as CT can be helpful especially in differentiating CH from other masses such as soft tissue sarcomas. CT is also useful in determining the extent of the lesion before surgery, but sometimes CT can be non-diagnostic, probably due to the absence of a formal cystic wall [8].

MRI provides precise preoperative staging, which is crucial for identifying the individual cystic loci, it can determine the extent of involvement of near structures, most importantly nerves and vessels, thus providing beneficial information for the surgeon, and therefore, MRI is encouraged in every suspected case of CH [9].

Axillary cystic hygroma is rare and less frequent than cervical, cases reported in the literature were diagnosed in the prenatal period during routine U/S tests, or in adulthood, we were not able to find any reported case in the pediatric age group, so we considered reporting this case.

These tumors typically develop slowly, and spontaneous regression is unusual, in this case, it developed rapidly in three days, Michail [2] also described this pattern of development, as he reported a case of axillary cystic hygroma which appeared in 12 h with the absence of any predisposing factor.

Management of choice of CH is still surgical incision, but other modalities have also shown success, these include sclerosing agents such as OK432 or fibrin adhesive [6], aspirations, radiation, laser excision, radio-frequency ablation, and cauterization [10]. In this case, we approached surgical excision due to the patient and the patient's parents' preference of removing this mass with its rapidly enlarging nature which bothered him and raise concerns of malignancy, also confirmed our diagnosis with histopathology.

## 4 Conclusion

Cystic hygroma occurs due to a malformation in the lymphatic system that causes a lack of communication with the venous system, as a result, cystic spaces form which can be unilocular or multilocular, these cysts become filled with lymphatic fluid that can't reach the venous system, which causes swelling. CH is a condition in children under two years of age, it typically occurs in the cervicofacial region, but can arise anywhere in the body. Workup with appropriate imaging measures is necessary to exclude other possible diagnoses and to confirm the diagnosis of cystic hygroma, management of choice is complete surgical excision, but other treatment choices can be also used as a cure or as a temporary solution.

The present case suggests that although unusual in the pediatric age group, CH should be considered in the differential diagnosis of any cystic axillary swelling.

## Consent

Written informed consent was obtained from the patient's parents for publication of this case report and accompanying images. A copy of the written consent is available for review by the Editor-in-Chief of this journal on request.

## Provenance and peer review

Not commissioned, externally peer reviewed.

## Ethical approval

The study is exempt from ethical approval in our institution.

## Funding

No funding or grant support.

## Guarantor

Dr. Rafiq Salhab.

## Research registration number

Not applicable.

## CRediT authorship contribution statement

Study concept or design: Rafiq Salhab
Writing the manuscript: Layth Al-Karaja, Mohammad G. Ibdah, Salem M. Tos, Narmeen Giacaman, Ahmadullah Musleh Abu Yousef
Review & editing the manuscript: Layth Al-Karaja, Mohammad G. Ibdah.

## Declaration of competing interest

There is no conflict of interest.
